# Prevalence of periodontitis in obese patients in Bahrain: a cross-sectional study

**DOI:** 10.1186/s12903-021-01720-y

**Published:** 2021-07-24

**Authors:** Leena Alsalihi, Crawford Bain, Alexander Milosevic, Amar Hassan, Abeer Janahi, Gowri Sivaramakrishnan

**Affiliations:** 1 Dental Postgraduate Training Department, Ministry of Health, Manama, Bahrain; 2 Periodontics Department, Hamdan Bin Mohammed College of Dental Medicine, Mohammed Bin Rashid University of Medicine and Health Sciences, Dubai, UAE; 3 Prosthodontics Department, Hamdan Bin Mohammed College of Dental Medicine, Mohammed Bin Rashid University of Medicine and Health Sciences, Dubai, UAE; 4 Hamdan Bin Mohammed College of Dental Medicine, Mohammed Bin Rashid University of Medicine and Health Sciences, Dubai, UAE; 5 Dental and Oral Health Services Department, Ministry of Health, Manama, Bahrain

**Keywords:** Overweight, Bodyweight, Obese, Periodontal disease

## Abstract

**Background:**

Adult obesity has been associated with various systemic diseases and is an increasing problem in Bahrain. Recent evidence indicates a correlation between adult obesity and periodontitis. Hence the aim of this study was to assess the prevalence of periodontitis in overweight/obese adults in Bahrain and to determine the factors associated with periodontitis in these obese adults.

**Method:**

This cross-sectional study was conducted in overweight subjects attending Ministry of Health (MOH) Nutrition Clinics at primary health centers in Bahrain. After obtaining the institutional ethics approval, the demographic and anthropometric data, including Body Mass Index (BMI) and waist circumference (WC) using World Health Organization (WHO) thresholds for severity of obesity, were recorded. Periodontal status was measured using the Community Periodontal Index (CPI) and the extent and severity of periodontal disease were categorized according to the number of sextants with CPI codes 3 and 4.

**Results:**

A total of 372 participated with a mean age 44.0 (± 10.5) years for males, and 42.5 (± 11.2) years for females. Periodontitis was present in 361 (97%) of participants. Hypertension and diabetes were the most prevalent co-morbidities at 23.4% and 16% respectively. Mean WC was significantly greater in males at 114 cm (± 15.6) compared to females 109.5 cm (± 12.5) (*p* < 0.001). BMI was not associated with severity or extent of periodontitis but WC was weakly correlated in males but not in females (Spearman rho =  + 0.2, *p* < 0.05). In the logistic regression model using overall WC to predict the severity of periodontitis, the adjusted OR was 1.02 (95% CI 1.00–1.04) and for age it was 1.05 (95% CI 1.00–1.07).

**Conclusion:**

The prevalence of periodontitis was high in this sample of overweight Bahrainis. BMI was not correlated with periodontitis but WC had a weak positive correlation. Implementation of periodontal health screening as a routine part of a nutrition clinic program is recommended.

## Background

Adult obesity is a global problem and has been on the rise. About 13% of the world’s adult population were reported to be obese in 2016 [1]. The World Health Organization (WHO) reported that obesity leads to chronic health outcomes, such as diabetes, cardiovascular diseases, musculoskeletal disorders and some cancers [2, 3]. Obesity is also a major risk factor for increased susceptibility to periodontal disease and is likely to have a substantial population-attributable risk [3]. Suvan et al. [4] reported that periodontitis was more prevalent in adult obese patients. Many other systematic reviews and meta-analyses in the past explored the association between obesity/ overweight and periodontitis identified obesity as a risk factor for periodontal disease [5–7].

**Fig. 1:**
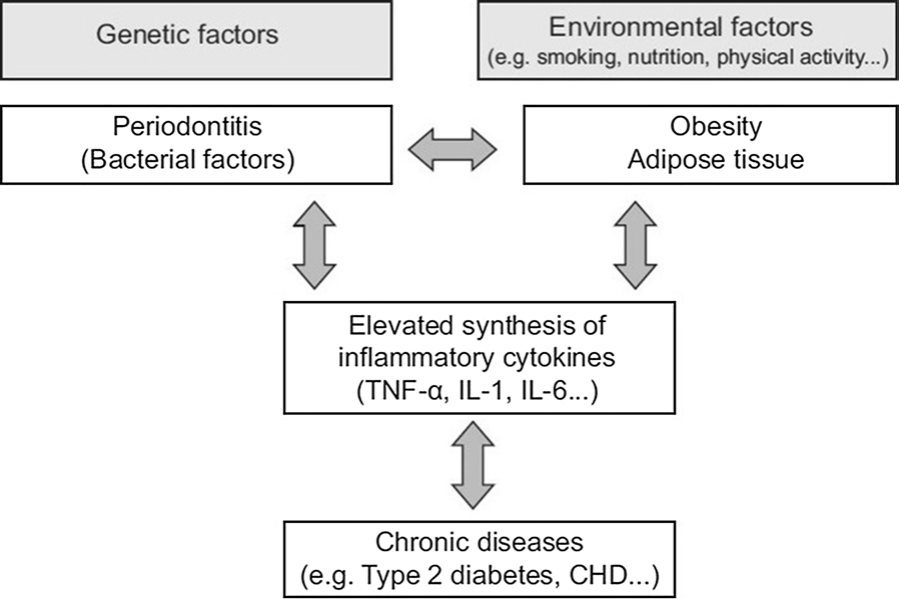
Model linking periodontitis, and obesity with inflammatory related chronic diseases

Obesity is a chronic low-grade inflammatory disease where the adipocytes secrete dozens of biologically active molecules such as leptin, resistin, tumor necrosis factor α (TNF-α), interleukins:IL-1, IL-6, IL-8 and IL-10, growth factors, complement components, angiotensinogen, plasma plasminogen, activator-1(PAI-1), and a number of other substances. This leads to alteration of the host response to antigens derived from bacterial plaque, thus causing periodontitis [8, 9] (Fig. 1). Specifically, the salivary IL-6 levels were significantly elevated in patients with periodontitis [10]. Recent advances and studies have also identified other salivary biomarkers such as Galectin as a potential modulator of several biological functions and inflammatory processes, including periodontitis [11]. The relationship between Galectin and obesity has been studied, which revealed that Galectin can be considered a potential therapeutic target in obesity and diabetes [11]. Recent evidence by Isola et al. [12] also revealed that Galectin and endothelin were significantly evident in patients with periodontitis with or without cardiovascular diseases. Another similar study also reported elevated levels of soluble urokinase-type plasminogen activator receptor (suPAR) in patients with periodontitis with or without cardiovascular diseases [13]. This explains the importance and role of the above mentioned salivary biomarkers in inflammatory processes like obesity and cardiovascular diseases. These are all found to be elevated in periodontitis patients. It is important to note that “host modulation therapy” which is the main stay of pharmacotherapy in periodontitis modulates the host response by inhibition or resolution. The host response is primarily based on the molecules and markers mentioned above [14].

BMI (Body Mass Index) and Waist circumference (WC) have traditionally been used to measure and diagnose underweight and overweight [15–17]. Prevalence of adult obesity in Bahrain is increasing at an alarming rate, with associated morbidity and mortality [18]. Most adults in primary care are overweight or obese; two thirds of patients with weight problems have other obesity-related conditions [18, 19]. Considering this association between obesity, periodontal disease and other diseases, there is a need for prevention. Hence, the aim of this study was to investigate the prevalence of periodontitis in obese adults in the Kingdom of Bahrain, and assess which measure of obesity (BMI, WC) is a better predictor for periodontitis.

## Method

### Study design, location and population sample

A convenience sample of obese/overweight participants were recruited from Five Nutrition Clinics at Primary Health Centers, Ministry of Health in Bahrain during the six-month study period in a cross-sectional study. This study was conducted in full concordance with the principles of the Declaration of Helsinki, Good Clinical Practice (GCP). Informed consent was obtained from all the participants of the study. Ethics approval was obtained from the primary care ethics committee, Training Affairs, Ministry of Health, Bahrain, (reference number MA/JE/647/2016).

### Sample size and technique

The sample size was calculated based on the sample used in previous similar studies and the number of registered obese patients in obesity clinics in Bahrain. The calculated sample size was 354 using the Cochran’s sample size calculation for cross-sectional design, with error 0.05 and estimated 20% of nonresponse rate. A convenience sampling method was used.

### Inclusion and exclusion criteria

Adult patients, ≥ 18 years old, with a minimum of 20 natural teeth excluding wisdom teeth, were included. Pregnant women, patients on chronic non-steroidal anti- inflammatory drug therapy and/or antibiotic use in the last 6 months were excluded.

### Data collection

Data was collected using a standardized data extraction sheet. Once the data sheet was checked for completeness, the sheets were coded to maintain anonymity. Information regarding the participants’ personal data, education status, medical history, health related behavior (smoking, alcohol drinking) as well as anthropometric measurements (BMI, WC, height and weight) were recorded by the first author of the study.

### Periodontal examination

Periodontal examination of participants was performed using the WHO Community Periodontal index (CPI) by the first author of the study. The five CPI scores were [19]:

- CPI 0 = Normal.
- CPI 1 = gingival bleeding.
- CPI 2 = calculus/plaque retentive factors
- CPI 3 = shallow periodontal pocket of 3.5-5.5mm.
- CPI 4 = deep periodontal pocket of > 5.5 mm.

The measurements were made using a WHO periodontal probe (E- probe) at six sites (Mesiobuccal, Midbuccal, Distobuccal, Mesiolingual, Midlingual, Distolingual) per tooth. Ten teeth were selected for the periodontal examination; two molars (excluding the third molar) in each posterior sextant and the upper right and lower left central incisors in the anterior sextants. If the index teeth were not present in a qualifying sextant, the adjacent remaining teeth in the sextant was examined. When a code 4 was recorded, no further probing in that sextant was done and a diagnosis of periodontitis was made. The presence or absence of periodontitis was dichotomized, and the subjects were categorized into two groups.

- Periodontitis absent = CPI 0 to CPI 2 (healthy periodontal tissues, gingivitis, and or plaque retentive factors)
- Periodontitis present = CPI 3 or CPI 4 (probing depth > 3.5 mm)

Periodontitis was considered severe if the individual CPI score was 4. Mild-moderate if the individual CPI score was 3. The number of sextants with periodontitis (CPI 3 or CPI 4) was also recorded per subject. Periodontitis was considered mild if the number of sextants with CPI 3 & 4 was 1 or 2, moderate if the number of sextants with CPI 3 & 4 was 3–4, and severe if number of sextants with CPI 3 & 4 was 5 or 6.

### Assessment of obesity

The clinical assessment of BMI, WC, Weight and Height was measured electronically using a special scale (Seca GmbH & Co KG, Germany) by the first author of the study. The WHO classification (2000) was used to define the degrees of overweight [20]:

- Overweight if BMI ranges 25.0–29.9 (kg/m^2^)
- Obese if BMI ≥ 30.0, sub-divided into:
  - Class I (moderate obese): BMI 30.0–34.9 (kg/m^2^).
  - Class II (severe obese): BMI 35.0–39.9 (kg/m^2^).
  - Class III (very severe obese): BMI > 40.0 (kg/m^2^).

Two gender specific waist circumference categories were used to classify WC into high or normal. The cut-off point or threshold of > 94 cm (> 37 inches) for male and of > 80 cm (> 32 inches) for female was selected according to the WHO classification, 2008 [15].

## Statistical analysis

The collected data were analyzed using the Statistical Package for Social Sciences (SPSS, version 20, Chicago, SPSS Inc.). Descriptive statistics were used for demographic data. Chi-square and Exact Fisher test were used to identify the differences between categorical data and t-test was performed to compare continuous variables. ANOVA was performed to analyze categorical data with a continuous variable. Cross-tabulation and Pearson Chi-Square analyses were used to determine which obesity measure was associated with the severity and extent of periodontal disease. A stepwise regression model was performed to explain periodontitis as a function of different variables. The level of statistical significance was set at 5%.

## Results

### Demographic data

A total of 372 obese and overweight participants, aged between 18 and 70 years with a mean age of 42.9 years (SD) (± 11), were included. The BMI and WC calculation are presented in Table 1. Periodontitis (CPI 3& 4) was most prevalent in the 40–50 year age group. Significant severity was found if the obese participants were aged over 40 years (*p* = 0.012). Significantly more females had periodontitis than males (*p* = 0.001). The prevalence of hypertension was significantly greater in the CPI 4 group compared to CPI 3 group (*p* < 0.05), but none of the other medical conditions were significantly different (Table 2).

**Table 1:** Number of obese participants grouped by different categories of BMI and gender specific WC categories (WHO guideline)

Categories of BMI	Male N (%)	Female N (%)	Total N (%)
Overweight	16 (14.5%)	28 (10.7%)	44 (11.8%)
Obese I	49 (44.5%)	92 (35.1%)	141 (37.9%)
Obese II	22 (20.0%)	77 (29.4%)	99 (26.6%)
Obese III	23 (20.9%)	65 (24.8%)	88 (23.7%)
Total	110 (100%)	262 (100%)	372 (100%)

Gender WC categories	N (%)		
Male			
WC ≤ 94 cm	20 (5.4%)		
WC > 94 cm	105 (24.2%)		
Female			
WC ≤ 80 cm	4 (1.1%)		
WC > 80 cm	258 (69.4%)		
Total	372 (100%)		

Gender	Mean WC (SD)		
Male	114.945 (± 15.6)		
Female	109.523 (± 12.5)		
Overall	111.126 (± 13.7)

**Table 2:** Prevalence of periodontitis according to socio-demographic factors, habits, and oral and general health status of obese participants

Variables	Periodontitis		Total	*p* value
	CPI = 3	CPI = 4		
	N%	N%	N%	
Age (year)				
18–30	37 (16.6%)	18 (13.0%)	55 (15.2%)	0.012*
31–39	57 (25.6%)	22 (15.9%)	79 (21.9%)	
40–50	82 (36.8%)	49 (35.5%)	131 (36.3%)	
≥ 51	47 (21.1%)	49 (35.5%)	96 (26.6%)	
Total	223 (100%)	138 (100%)	361 (100%)	
Gender				
Male	54 (24.2%)	55 (39.9%)	109 (30.2%)	0.001*
Female	169 (75.8%)	83 (60.1%)	252 (69.8%)	
Total	223 (100%)	138 (100%)	361 (100%)	
Marital status				
Single	18 (8.1%)	12 (8.7%)	30 (8.3%)	0.490
Official Married*	205 (91.9%)	126 (91.3%)	331 (91.7%)	
Total	223 (100%)	138 (100%)	361 (100%)	
Occupation				
Employed	107 (48.0%)	74 (53.6%)	181 (50.1%)	0.375
Unemployed*	32 (14.3%)	22 (15.9%)	54 (15.0%)	
House wife	84 (37.7%)	42 (30.4%)	126 (34.9%)	
Total	223 (100%)	138 (100%)	361 (100%)	
Education status				
Intermediate &less*	39 (17.5%)	30 (21.7%)	69 (19.1%)	0.570
Secondary	88 (39.5%)	54 (39.1%)	142 (39.3%)	
University	96 (43.0%)	54 (39.1%)	150 (41.6%)	
Total	223 (100%)	138 (100%)	361 (100%)	
Smoking	18 (8.1%)	13 (9.4%)	31 (8.6%)	0.396
Tooth brushing frequency				
< 1 time/day	12 (5.4%)	7 (5.1%)	19 (5.3%)	0.552
≥ 1/day	211 (94.6%)	131 (4.9%)	342 (94.7%)	
Total	223 (100%)	138 (100%)	361 (100%)	
Medical conditions				
Hypertension	46 (20.6%)	40 (29.0%)	86 (23.8%)	0.047*
Heart disease	8 (3.6%)	4 (2.9%)	12 (3.3%)	0.488
Diabetes Mellitus	32 (14.3%)	27 (19.6%)	59 (16.3%)	0.124
Hyperlipidemia	32 (14.3%)	16 (11.6%)	48 (13.3%)	0.280
Endocrine system	14 (6.3%)	8 (5.8%)	22 (6.1%)	0.523
Other	21 (9.4%)	9 (6.5%)	30 (8.3%)	0.222

*Officially married: Married, Divorced, Widowed*Unemployed: Student, Retired, Unemployed*Intermediate &less: illiterate, primary, intermediate

### Prevalence and severity of periodontitis in relation to BMI and WC

The prevalence of periodontitis was extremely high (97%) in obese patients. 59.9% of obese patients had a CPI score 3(mild- moderate periodontitis) and 37.1% had CPI score 4 (severe periodontitis). Analysis of the relationship between categories of BMI and severity of periodontitis did not reveal any significant differences between the groups (*p* = 0.743) (Table 3). However, severe periodontitis was weakly associated in males (N = 49) with a WC greater than the threshold of 94 cm (Spearman rho =  + 0.2). The WC in females was not associated with the severity of periodontitis (Table 3).

**Table 3:** Prevalence and severity of periodontitis according to categories of BMI and gender specific WC by using WHO guideline

Variables	Periodontitis		Total	*p* value
	CPI = 3	CPI = 4		
	N%	N%	N%	
Categories of BMI				
Overweight	28 (12.6%)	13 (9.4%)	41 (11.4%)	0.743
Obese I	86 (38.6%)	52 (37.7%)	138 (38.2)	
Obese II	59 (26.5%)	37 (26.8%)	96 (26.6%)	
Obese III	50 (22.4%)	36 (26.1%)	86 (23.8%)	
Total	223 (100%)	138 (100%)	361 (100%)	
Gender WC categories				
Male				
WC ≤ 94	14 (25.9%)	6 (10.9%)	20 (18.3%)	0.037*
WC > 94	40 (74.1%)	49 (89.1%)	89 (81.7%)	
Total	54 (100%)	55 (100%)	109 (100%)	Spearman rho = + 0.2
Female				
WC ≤ 80	3 (1.8%)	1 (1.2%)	4 (1.6%)	0.600
WC > 80	166 (98.2%)	82 (98.8%)	248 (98.4%)	
Total	169 (100%)	83 (100%)	252 (100%)	

^*^p < 0.05. CPI score 3 = mild-moderate periodontitis, CPI score 4 = severe periodontitis

Table 4 shows the extent of periodontitis determined by the number of sextants with CPI 3 or 4. There were no significant differences in the distribution of participants by WHO BMI category and the extent of periodontitis, but the overall mean WC was high in patients that presented with CPI 3 and 4 in all the sextants. (ANOVA = 2.767, *p* < 0.05) (Table 4).

**Table 4:** Extent of periodontitis by number of sextants with CPI 3 and 4 per participant according to categories of BMI and overall WC mean

Categories of BMI	Extent of Periodontitis(CPI 3 and 4) sextants N%			Total	*p* value
	1–2 sextants	3–4 sextants	5–6 sextants		
Overweight (25–29.9)	6 (10.3%)	30 (15.1%)	5 (4.8%)	41 (11.4%)	0.161
Obese class I (30–34.9)	27 (46.6%)	74 (37.2%)	37 (35.6%)	138 (38.2)	
Obese class II (35–39.9)	14 (24.1%)	49 (24.6%)	33 (31.7%)	96 (26.6%)	
Obese class III (≥ 40)	11 (19.0%)	46 (23.1)	29 (27.9%)	86 (23.8%)	
Total	58 (100%)	199 (100%)	104 (100%)	361 (100%)	

Extent of Periodontitis (CPI 3 and 4) by number of affected sextants	Number of participants	Overall WC Mean (SD)	*p* value
1–2 sextants	58	109.4 (12.9)	0.042*
3–4 sextants	199	110 (14.1)	
5–6 sextants	104	114.3 (12.9)	
Total	361(100%)	111.2 (13.7)	

*Signifies that the *p* value is significant

A stepwise logistic regression model to explain periodontitis as a function of different variables is shown in Table 5. The fitness of the model is significant (*p* < 0.001), and the severity of periodontitis was explained 11% by the overall WC and age adjusted for gender and blood pressure, the overall WC OR was 1.02 with 95% CI (1.00–1.04) and for age it was 1.05 with 95% CI (1.00–1.07).

**Table 5:** Logistic regression to explain periodontitis on function of different variables

Variables	B	S.E	Wald	*df*	Sig	OR (95% CI)
WC	0.021	0.009	5.334	1	0.021	1.021 (1.003–1.039)
Gender	− 0.433	0.257	2.843	1	0.092	0.648 (0.392–1.073)
Age	0.047	0.012	14.546	1	0.000	1.048 (1.023–1.074)
BP	0.039	0.290	0.018	1	0.893	1.040 (0.589–1.834)
Constant	− 4.615	1.302	12.573	1	0.000	0.010

## Discussion

The present study aimed to identify the prevalence and severity of periodontitis in obese adults in Bahrain. A total of 372 participated with a mean age 44.0 (± 10.5) years for males, and 42.5 (± 11.2) years for females. Periodontitis was present in 361 (97%) of participants with the highest prevalence in the 40–50 year age group. Hypertension and diabetes were the most prevalent co-morbidities at 23.4% and 16% respectively. Mean WC was significantly greater in males at 114 cm (± 15.6) compared to females 109.5 cm (± 12.5) (*p* < 0.001). Our study results indicate that BMI was not associated with severity or extent of periodontitis but WC was weakly correlated in males but not in females. Similar studies on obese patients in a sample of the population reported that the prevalence of chronic periodontitis was 35–70% [21–24].

Periodontitis was significantly more prevalent in the 40–50-year-old obese participants in our study. However, this is in contrast to previous studies that reported higher prevalence of periodontitis in younger obese adults [22, 25]. Evidence suggests that ageing is commonly associated with periodontal disease [26]. This can possibly be attributed to the cumulative periodontal breakdown with age. In general, as we age, body fat increases whereas lean mass and bone mineral density decrease. Another major change is that fat mass tends to be preferentially distributed in the abdominal region, a phenomenon that has been reported in both sexes. This leads to obesity. Ageing is associated with progressive changes in total and regional fat distribution that have negative health consequences [26, 27]. Our study found that the severity of periodontitis was more pronounced in obese participants aged over 40 years. Moreover, the logistic regression model found that the severity of periodontitis can be marginally predicted by age.

In our study, periodontitis was more prevalent in obese female participants (69.8%). This finding was similar with the findings of the study conducted by Khan et al. [23] on obese Malaysian participants, who reported a prevalence rate of 61.3% in obese females. In addition, Dalla Vecchia et al. [28] found that obese females had an 80% higher chance of having periodontitis. This can be attributed to the changes in hormonal levels of estrogen and progesterone during pre-menstrual and menstrual phases that induces the production of inflammatory cytokines in the periodontium, leading to periodontitis [29].

The present study found that the prevalence of hypertension was significantly greater in the CPI 4 group compared to the CPI 3 group, but none of the other medical conditions were significantly different (*p* < 0.05). The current study also found that the prevalence of periodontitis was not associated with tooth brushing frequency (*p* = 0.579).

In the present study, we found that the degree of obesity using the WHO guidelines had no significant association with the prevalence or the severity of periodontitis in this sample of obese Bahraini participants (*p* = 0.743). Moreover, the extent of periodontitis (the number of sextants with CPI 3 or 4) was found to have no significant association with the degree of BMI (*p* = 0.161). In disagreement with our results, two recent studies on obesity found that an increase in BMI was associated positively with periodontal disease [21, 22].

The current study found that there is a significant relationship between the number of sextants with CPI 3 or 4 and the overall mean WC in obese participants (*p* = 0.042). Similar results were reported from previous studies by Kim et al. [30], Al Zahrani et al. [22] and Khader et al. [21]. Moreover, our finding suggested that obese males were significantly associated with the greater severity and extent of periodontitis. The finding of the significant association between the severity of periodontitis and WC in males as compared to females might be due to the fact that men have more body fat in the abdominal (visceral) region than women [31].

There is a significant body of evidence to support the association between periodontal disease and other NCD’s (Non-communicable diseases) including cardiovascular diseases which accounts for almost 45% of NCD induced mortality [32]. This is related to the rise in the inflammatory burden as seen in obesity and periodontitis that is discussed in this study. Diets that are high in saturated fats, salt and refined sugars significantly contribute to obesity and type 2 diabetes mellitus. This is a major attributable risk factor for myocardial infarction and other CVDs [33]. This study result focusses on the need to educate patients on the link between oral health and CVD.

Our data suggest a weak positive correlation of WC and the severity of periodontitis in males, whereas there was no correlation with BMI. Thus the present study concludes that the overall WC mean is a predictor of the severity of periodontitis, irrespective of the gender. The limitations of the study were that a cross sectional study design does not allow us to infer relationships. A longitudinal cohort study design in the future would provide more conclusive evidence. A convenience sampling technique was used which can incorporate bias. The absence of a non-obese control group limited our ability to assess any association between obesity and periodontal disease. Partial recording of periodontal disease has been shown to have reliability, good utility and high sensitivity whilst the 2018 case definition has improved the precision performance on ‘CPITN’ teeth using a 6-point CPITN pocket measurement, although underestimation of periodontitis has been reported regarding pocket depth estimation and mean clinical attachment loss [34].

## Conclusion

Within the limitations of the study, WC measurement in obese patients can be effectively used as a predictor of prevalence and severity of periodontal disease. This study reflects the periodontal status of a non-randomised convenience sample of obese Bahraini adults and highlights the effect of obesity on periodontal health. The introduction of periodontal health screening as a routine part of the nutrition clinic program in MOH/Bahrain is recommended as is the implementation of oral hygiene advice in obese/overweight Bahrainis.

## Data Availability

The datasets used and/or analyzed during the current study are available from the corresponding author on reasonable request.

## Abbreviations

BMI: Body Mass Index
WHO: World Health Organization
WC: Waist circumference
CPI: Community periodontal index
GCP: Good Clinical practice
